# The Establishment of Weak Ecological Microexpressions Recognition Test (WEMERT): An Extension on EMERT

**DOI:** 10.3389/fpsyg.2019.00275

**Published:** 2019-03-05

**Authors:** Ming Yin, Liangchen Tian, Wei Hua, Jianxin Zhang, Dianzhi Liu

**Affiliations:** ^1^Jiangsu Police Institute, Nanjing, China; ^2^School of Humanities, Jiangnan University, Wuxi, China; ^3^School of Education, Soochow University, Soochow, China

**Keywords:** weak ecological microexpression recognition test, backgrounds, JACBART microexpressions, fluctuation of weak microexpression recognition, openness

## Abstract

The JACBART (Japanese and Caucasian Brief Affect Recognition Test) microexpression recognition test only examines facial expressions under the neutral expression background and the ecological validity is not high. The EMERT (Ecological MicroExpressions Recognition Test) microexpression recognition test examined six microexpressions under seven backgrounds but does not detect the intensity of expressions. In the current study, a weak ecological microexpression recognition test was established to examine the recognition features of six weak microexpressions in all seven high intensity basic expressions. The results found: (1) the test had good retest reliability, criterion validity and ecological validity; and (2) the reliability and validity tests revealed a lot of characteristics of weak microexpression recognition. There were training effects in some weak microexpression recognition. Weak microexpression recognition was generally positively related to the microexpression recognition of JACBART but were generally negatively related to approximate common expressions. The backgrounds main effects in all weak microexpressions were significant and pairwise comparisons show there were a wide range of differences between weak microexpressions under different backgrounds. The standard deviations, of the accuracy of weak microexpressions in different backgrounds, were used to define the fluctuations of the weak microexpression recognition and we found that weak microexpression recognition had many fluctuations. (3) Personality openness and its subdimensions (O1, O2, O3, and O5) were generally positively related to some weak microexpression recognition, except O1, which was significantly negatively related to surprise under neutrality. O1 was positively related to the standard deviation of the weak anger microexpression recognition accuracies and O6 was negatively related to the standard deviation of the weak happiness microexpression recognition accuracies in the first measurement.

## Introduction

### JACBART Microexpression Recognition Test

Microexpressions are very transitory expressions lasting about 1/25–1/2 s, which can reveal people’s true emotions that they tend to hide or suppress (Ekman and Friesen, 1975; Porter et al., 2012). Therefore, microexpressions, as an important tool to detect true feelings, can be applied in the field of lie detection and clinical psychological areas (Wu et al., 2010; Hurley and Frank, 2011). Matsumoto et al. (2000) developed the Japanese and Caucasian Brief Affect Recognition Test (JACBART) to measure microexpression recognition. The participants would first see a neutral image for 2000 ms, then microexpressions were presented for a short time, followed by a neutral image for 2000 ms again. Participants needed to check out the microexpression type. The neutral image before and after the microexpressions could eliminate the visual aftereffects of the microexpressions. Researchers that used the JACBART found that people could easily recognize the common expressions, but recognition of the microexpressions proved difficult with accuracies usually between 45 and 59% (Hall and Matsumoto, 2004; Russell et al., 2006; Matsumoto and Hwang, 2011).

### Ecological Microexpression Recognition Test (EMERT)

The JACBART paradigm only used neutrals to eliminate the visual aftereffects of the microexpressions, but it did not examine the influence backgrounds had on emotional expressions. Therefore, Zhang et al. (2014) explored for the first time the background effects on microexpressions and found that when backgrounds were negative (sad), all microexpressions (anger, disgust, fear, surprise, and happiness) recognition accuracies were significantly lower than those with positive (happy) or neutral backgrounds; when the backgrounds and the microexpressions were consistent in property (negative or positive), microexpression recognition accuracies were significantly lower than that when they were inconsistent in property. The research has shifted the JACBART paradigm. Though it is very instructive, it need to be developed further: (1) It does not explore all backgrounds or microexpressions; (2) it does not reveal that microexpressions in different backgrounds are ecological microexpressions and it does not set up an ecological microexpression recognition test to test reliability and validity; (3) it does not examine intensity factors such as valence or arousal in backgrounds and microexpressions.

Yin et al. (2016) proposed for the first time that all basic expression types, for both backgrounds and microexpressions, needed to be detected in order to set up an ecological microexpression recognition test. Therefore, Zhang et al. (2017) examined the recognition characteristics of six basic expression types of microexpressions (sadness, aversion, fear, anger, neutrality, surprise, happiness) under seven basic expression types of backgrounds (the six basic expression types and neutrality), to establish an ecological microexpression recognition test—EMERT (Ecological MicroExpressions Recognition Test) and found that the test had good retest reliability, criterion validity and ecological validity: (1) the ecological microexpression recognition was generally significantly related to the JACBART microexpression recognition and common expression recognition; (2) the backgrounds main effect of fear, sadness, disgust and anger microexpressions were significant, while the backgrounds main effects of surprise and happiness microexpressions were not significant, but there was a large difference between them in common expressions; (3) The ecological microexpression recognition had stable fluctuation. The current study however, found that EMERT did not examine intensity factors such as valence or arousal, because both its backgrounds and microexpressions were of the same high intensity–the intensity (combination of valence and arousal) four expressions of Ekman and Friesen’s (1976) international expression database.

### Personality Influence Factors of Microexpression Recognition

Matsumoto et al. (2000) used JACBART and found that the microexpression recognition ability was positively related to extroversion and conscientiousness in the five personality factors. Mill et al. (2009) found that people with high openness and responsibility were better at identifying microexpressions. Hurley et al. (2014) found that college students with high openness had a stronger ability to identify microexpressions. But these studies used JACBART and did not study how personality influenced ecological microexpression recognition ability.

### Shortcomings of Previous Studies and Improvements Made in the Current Study

Zhang et al. (2014) used sadness, neutrality and happiness as backgrounds, but did not explore all types of microexpression recognition under all backgrounds. Furthermore, they did not establish a standardized test and did not examine the intensity factor. Zhang et al. (2017) used all seven basic expressions as backgrounds and six basic expressions as microexpressions to EMERT, but its backgrounds and microexpressions, still of the same high intensity 4, did not examine the intensity factor. Therefore, ecological microexpressions in EMERT represent a special case of ecological microexpressions and real microexpressions, because in real life both backgrounds and microexpressions may be of low and high intensity.

Therefore, the current study used seven high intensity basic expressions (sadness, aversion, fear, anger, neutrality, surprise, happiness, and the intensity of expressions except neutral is 4) as backgrounds and six low intensity basic expressions (the intensity is 2) except neutrality which is embedded between the high intensity backgrounds as a weak microexpression. The current study therefore establishes, for the first time, a weak microexpression recognition test and detects its reliability and validity, a type of ecological microexpression recognition test, but its microexpression intensity is lower than that used by Zhang et al. (2017)’s EMERT. It can therefore be called the weak ecological microexpression recognition test (WEMERT).

Some concepts need clarification: (1) the real microexpressions are those backgrounds and target microexpressions that include three main dimensions, such as expression type (sadness, aversion, fear, anger, neutrality, surprise, happiness), intensity (valence and arousal) and time, and in the process of a real microexpression, the expression type and intensity changes with time; (2) the ecological microexpressions are the experimental approximation of the real microexpression, so they investigate the three main dimensions mentioned above in backgrounds and microexpressions; (3) weak microexpressions are a type of the ecological microexpression; (4) the JACBART microexpressions are also a special case of the ecological microexpressions and their backgrounds are neutral; (5) background and microexpression are operational definitions. In the experimental paradigm of an ecological microexpression recognition test, the microexpressions are embedded in the foregrounds and backgrounds. The task asks the participants to judge the microexpression types. Microexpressions and backgrounds form the ecological microexpressions; (6) when the low intensity microexpressions and the high intensity backgrounds are of the same expression type, they are considered special weak microexpressions– approximate common expressions.

## Materials and Methods

### Participants

Ninety-eight male and female undergraduates and post-graduates from Soochow University were selected to participate in the study. Forty-nine participants had never participated in a microexpression recognition experiment before. The average age was 20.56 years old and the standard deviation was 2.01. All participants were right-handed with normal eyesight and without color blindness. They all volunteered and could quit the experiment at any time and all participants received a reward after completing the study. The experiments were approved by the Soochow University Education School’s Ethics Committee in China, in accordance with the ethical guidelines of the Declaration of Helsinki.

### Experimental Apparatus and Materials

Compared to EMERT (Zhang et al., 2017), the current study used similar experimental apparatuses and materials. Seven types of basic expression pictures, of 10 Caucasians (four males and six females) from the international expression database established by Ekman and Friesen (1976) were used as backgrounds, namely, neutrality, anger, disgust, fear, happiness, sadness, and surprise. Except for neutral expressions, the emotional intensity level of the other six types of expressions are 4. Emotional intensity is a combination of emotional valence and arousal as established by Ekman and Friesen (1976), and its levels range from low (1) to high (6), of which 4 is the highest emotional intensity level in true expressions, and 5 and 6 are higher levels as exaggerated by a computer. Except for neutral expressions, the other six types of expressions were used as weak microexpressions, with an emotional intensity level of 2 (the only difference from EMERT, whose microexpression emotional intensity level is 4). The images used were from the international expression database (Ekman and Friesen, 1976), and did not display any ears or hair. The shadow of facial expressions and head postures were the same in all images. The pixels of all images were modified to 338 × 434 and had a gray background (GRB: 127, 127, 127) (Zhang et al., 2017). Matsumoto et al. (2007) found that the seven basic expressions in 27 different countries were universal. We therefore used images of Caucasian expressions to measure microexpression recognition in Chinese college students. The Lenovo desktop computer M400-D003 and 19-inch CRT monitor with 1600 × 1200 resolution and 75 Hz refresh rate and a gray background was used to conduct the experiments. The E-prime 2.0 was used to compile the experimental procedure.

Zhang et al. (2003) revised the NEO-PI-R (Costa and McCrae, 1992) China version, which was divided into five dimensions, each containing six sub dimensions, and five-point scoring. Dai et al. (2004) found that the internal consistency reliability of the five dimensions, was between 0.77 (agreeable) and 0.92 (neuroticism); retest reliability was between 0.81 (open) and 0.91 (extrovert); and a factor analysis found that the structure validity was good; while a correlation analysis found that calibration validity was good using the Eysenck personality scale (EPQ) as calibration. The current study used the Openness sub scale, including six dimensions such as fantasy, beauty, feelings, actions, ideas and values, and each subdimension included eight questions.

### Experimental Design and Procedures

The current study used the EMERT experimental paradigm (Zhang et al., 2017), but placed low intensity microexpressions between two high intensity backgrounds to create a WEMERT. The experiment was 7 (high intensity backgrounds) × 6 (weak microexpressions) × 2 (two measurements) within the subject design. We chose expressions of neutrality, fear, sadness, disgust, anger, surprise, and happiness with an intensity level of 4 as backgrounds, with a presentation time was 800 ms. We chose expressions of fear, sadness, disgust, anger, surprise and happiness with an intensity level of 2 as weak microexpressions, with a presentation time of 133 ms (Matsumoto et al., 2000; Zhang et al., 2017). In one trial, foregrounds and backgrounds, and microexpressions were of the same model’s face and the foregrounds and backgrounds were the same. As there were seven types of expression backgrounds, in order to balance the sequential effect, the Latin square design was used to set up seven groups with seven females and males in each group. Each dependent variable in the seven groups was averaged in the results analysis.

Participants were 60 cm away from the screen. On the computer keyboard, six keys of SDF-JKL were labeled with ‘anger,’ ‘disgust,’ ‘fear,’ ‘sadness,’ ‘surprise,’ and ‘happiness.’ Before the experiment, the participants were asked to put the ring, middle and index finger of their left hand on the ‘angry,’ ‘disgust,’ and ‘fear’ keys, respectively, and the index, middle and ring finger of their right hand on ‘sadness,’ ‘surprise,’ and ‘happiness’ keys. Participants then completed a set of key pressing practice rounds. First, one of the six types of expressions (except neutrality) was presented for 1000 ms; then six labels “anger, disgust, fear, sadness, surprise, happiness” appeared on the screen, the participants needed to recognize it and press the right key as accurately as possible. There were 30 trials, and six types of expressions were pseudo-randomly presented five times.

**FIGURE 1:**
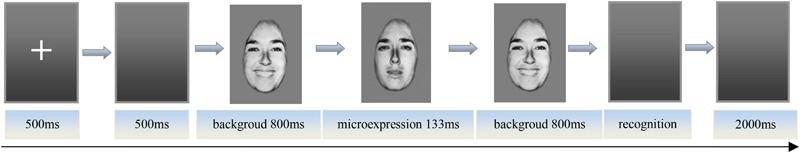
The picture of experiment procedure.

After the key pressing practice was completed, the instructor informed the participants of the procedure. First, the center of the screen showed the “+” for 500 ms; second, the empty screen lasted 500 ms; then the foreground expression picture was presented for 800 ms, after which the microexpression pictures would appear for 133 ms, followed by 800 ms of a background expression picture. The foreground was the same as the background. Participants needed to try to identify the briefly-presented microexpressions between foreground and backgrounds. Later, six labels “anger, disgust, fear, sadness, surprise, happiness” appeared on the screen. The labels on the screen were arranged in the same order as the labels on the keyboard. The participants were asked to press a key according to the microexpressions they saw, as accurately as possible instead of as soon as possible (no time limit). After the participants pressed the key, an empty screen appeared for 2000 ms. Then the fixation point “+” was presented for 500 ms and the next trial started. The experimental procedure is shown in Figure 1.

**FIGURE 2:**
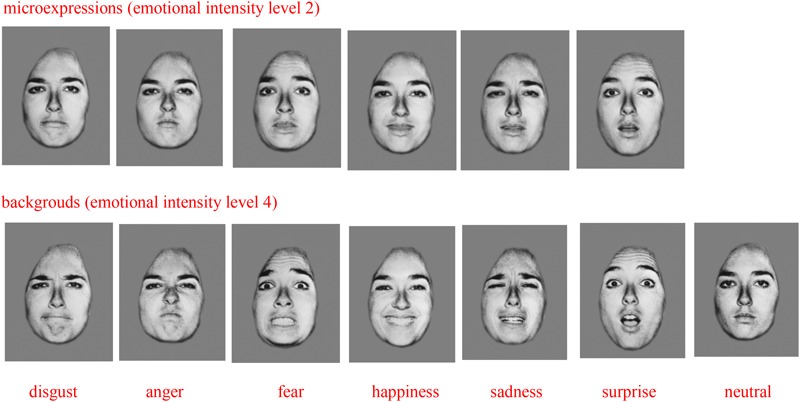
Some microexpressions and background images.

After receiving the instructions, participants practiced the experimental procedure. There was a total of 14 trials, in which seven types of backgrounds appeared twice, and six types of microexpressions each appeared two to three times. The participants were asked to determine the type of microexpression they observed. After the experimental procedure practice was completed, the screen showed “Are you clear about the task and operation of this experiment? If you are clear, press Q to start a formal experiment; if you have any questions, please ask the main test staff.” If the participants reported that they were clear, they started a formal trial; if they had any doubt, staff helped and instructed them to practice again. In order to allow the participants to get enough rest, the experiment was divided into seven blocks, one of the six types of expressions were chosen as a background for each block. One experiment therefore had seven (backgrounds) × 6 (microexpressions) × 10 (models) = 420 trials (Figure 2). A 2 min rest period was given between each two blocks.

In order to examine the retest reliability of the test, the participants needed to do two measurements, with a 1 week interval between each measurement. Before the second measurement, participants filled the openness subscale (Costa and McCrae, 1992; Zhang et al., 2003).

## Results

The SPSS 16.0 was used for the statistics. In the first measurement, 98 participants were included, 49 females and 49 males. In the second measurement, 97 participants were included, 48 males and 49 females. The establishment of WEMERT was not only the main purpose of the study, but also the premise of exploring its characteristics. Therefore, it was necessary to examine the reliability and validity of the test and reveal its characteristics in this process. The following indicators and order were adopted to examine the reliability and validity: (1) reliability: the correlation between the weak microexpression recognition in two experiments was taken as the retest reliability; (2) criterion validity: the weak microexpressions under neutrality were not only a special case of weak microexpressions, but also microexpressions of JACBART. The correlation between weak microexpression recognition under other backgrounds and under neutrality can be used as the criterion validity, indicating that weak microexpressions were a type of microexpression. When the background and microexpression were of the same expression type, for example, sadness, but the emotional intensity level of the background and microexpression were 4 and 2, what the participants saw was an approximate common expression, a special case of weak microexpressions. The correlation between the approximate common expression recognition and the other weak microexpression recognition can also be used as a criterion validity; (3) the first ecological validity: the main background effect of weak microexpression recognition and the difference between the weak microexpression recognition under different backgrounds, were ecologically valid; (4) the second ecological validity: the standard deviations of weak microexpression recognition under different backgrounds can be observed as a quantitative indicator of the backgrounds effect, which was used to measure fluctuations of weak microexpression recognition and as an ecological validity. It is worth noting that all microexpressions in the seven types of backgrounds were weak microexpressions, including the weak microexpressions under neutralitx and approximate common expressions, because in each of them, there were some difference in either the emotional intensity level or the expression type between the microexpression and background. The first characteristic of a weak microexpression recognition was the training effect reflected in the retest reliability analysis; the second characteristic of a weak microexpression recognition was the correlation among weak microexpressions reflected in the calibration validity; the third characteristic was the difference among different weak microexpression recognitions reflected in the first ecological validity analysis; the fluctuation of an ecological microexpression recognition, was reflected in the second ecological validity analysis; the fourth characteristic was the correlation between weak microexpressions and openness. In order to avoid repetition of the data analysis and discussion, these four characteristics and the corresponding reliability and validity were introduced together.

The accuracy and standard deviation of each weak microexpression in two measurements is shown in Table 1. Because the accuracy of the weak microexpression recognition in the second measurement might contain a training effect, the accuracy of that in the first measurement was taken as the weak microexpression recognition ability and as the dependent variable to conduct a 7 (backgrounds) × 6 (weak microexpressions) analysis of variance. Backgrounds and weak microexpressions were within-subject independent variables. (1) A sphericity test of backgrounds showed that the variance was not homogeneous, *p* < 0.05, we then applied a Greenhouse correction and found that the backgrounds main effect was significant, *F*(5.26,92) = 57.74, *p* < 0.001, $\eta_{p}^{2}$ = 0.373, which indicated that backgrounds affected weak microexpressions. (2) A sphericity test of weak microexpressions showed that the variance was not homogeneous, *p* < 0.05, we then applied a Greenhouse correction and found that the main effect of weak microexpressions was significant, *F*(3.84,93) = 157.97, *p* < 0.001, $\eta_{p}^{2}$ = 0.620, which showed that different weak microexpression recognitions was different. (3) A sphericity test of backgrounds × weak microexpressions showed that the variance was not homogeneous, *p* < 0.05, we the applied a Greenhouse correction and found that backgrounds and weak microexpressions had a significant interaction effect, *F*(5.07,87) = 21.93, *p* < 0.001, $\eta_{p}^{2}$ = 0.184, which showed that backgrounds and weak microexpressions influenced each other.

**Table 1:** The scores of weak microexpression recognition in two measurements and their relationship.

Weak microexpressions	The second measurement	The first measurement	Retest reliability	Paired simple *t-*test	Cohen’s *d*
	(*M* ±*SD, n* = 97)	(*M* ±*SD, n* = 98)	*r*(*df* = 96)	*t*(*df* = 96)	
Fear under fear	0.17 ± 0.26	0.18 ± 0.27	0.67**	-0.19	–
Sadness under sadness	0.26 ± 0.29	0.27 ± 0.26	0.61**	-0.29	–
Disgust under disgust	0.31 ± 0.26	0.30 ± 0.22	0.34**	0.44	–
Anger under anger	0.15 ± 0.21	0.17 ± 0.21	0.60**	-0.64	–
Surprise under surprise	0.24 ± 0.27	0.24 ± 0.27	0.60**	0.09	–
Happiness under happiness	0.17 ± 0.29	0.19 ± 0.31	0.58**	-0.63	–
Fear under neutral	0.13 ± 0.14	0.15 ± 0.17	0.30**	0.34	–
Fear under sadness	0.15 ± 0.17	0.15 ± 0.16	0.32**	0.26	–
Fear under disgust	0.14 ± 0.17	0.17 ± 0.17	0.39**	0.32	–
Fear under anger	0.14 ± 0.17	0.15 ± 0.16	0.15	0.34	–
Fear under surprise	0.09 ± 0.11	0.12 ± 0.13	0.39**	0.04*	-0.25
Fear under happiness	0.20 ± 0.19	0.19 ± 0.20	0.44**	0.25	–
Sadness under neutral	0.29 ± 0.22	0.27 ± 0.20	0.42**	0.28	–
Sadness under fear	0.22 ± 0.18	0.23 ± 0.21	0.47**	0.92	–
Sadness under disgust	0.19 ± 0.18	0.18 ± 0.18	0.32**	0.94	–
Sadness under anger	0.20 ± 0.18	0.18 ± 0.16	0.36**	0.28	–
Sadness under surprise	0.24 ± 0.18	0.20 ± 0.17	0.46**	0.03*	0.23
Sadness under happiness	0.34 ± 0.26	0.32 ± 0.23	0.50**	0.77	–
Disgust under neutral	0.46 ± 0.28	0.42 ± 0.25	0.40**	0.15	–
Disgust under fear	0.29 ± 0.21	0.24 ± 0.20	0.47**	0.54	–
Disgust under sadness	0.3 ± 0.23	0.27 ± 0.22	0.44**	0.95	–
Disgust under anger	0.24 ± 0.19	0.22 ± 0.16	0.34**	0.15	–
Disgust under surprise	0.33 ± 0.25	0.26 ± 0.24	0.46**	0.01**	0.29
Disgust under happiness	0.31 ± 0.23	0.27 ± 0.22	0.44**	0.16	–
Anger under neutral	0.22 ± 0.19	0.25 ± 0.18	0.46**	0.18	–
Anger under fear	0.22 ± 0.19	0.23 ± 0.22	0.46**	0.14	–
Anger under sadness	0.22 ± 0.19	0.22 ± 0.18	0.54**	0.58	–
Anger under disgust	0.15 ± 0.18	0.14 ± 0.16	0.30**	0.71	–
Anger under surprise	0.21 ± 0.22	0.22 ± 0.21	0.56**	0.28	–
Anger under happiness	0.22 ± 0.21	0.24 ± 0.21	0.66**	0.73	–
Surprise under neutral	0.68 ± 0.31	0.66 ± 0.28	0.50**	0.63	–
Surprise under fear	0.26 ± 0.19	0.26 ± 0.18	0.38**	0.38	–
Surprise under sadness	0.59 ± 0.30	0.56 ± 0.29	0.48**	0.33	–
Surprise under disgust	0.59 ± 0.30	0.56 ± 0.30	0.58**	0.00**	0.1
Surprise under anger	0.55 ± 0.27	0.47 ± 0.25	0.51**	0.63	–
Surprise under happiness	0.55 ± 0.30	0.51 ± 0.30	0.49**	0.02*	0.13
Happiness under neutral	0.68 ± 0.27	0.72 ± 0.26	0.54**	0.22	–
Happiness under fear	0.58 ± 0.32	0.59 ± 0.31	0.62**	0.32	–
Happiness under sadness	0.57 ± 0.27	0.56 ± 0.28	0.64**	0.22	–
Happiness under disgust	0.57 ± 0.30	0.57 ± 0.31	0.58**	0.25	–
Happiness under anger	0.69 ± 0.32	0.66 ± 0.34	0.59**	0.22	–
Happiness under surprise	0.65 ± 0.34	0.62 ± 0.32	0.58**	0.29	–

*^∗^*p* < 0.05, ^∗∗^*p* < 0.01.*

Since the participants had six keys to choose for each trial, the random level is 1/6. We made a single sample *t*-test for each weak microexpression recognition accuracy, with a random level of 1/6 and found that most of the weak microexpression recognition accuracies were significantly higher than random ones (*p*s < 0.05), which showed that the participants could effectively identify the weak microexpressions. But some of the weak microexpression recognition accuracies were not higher than the random ones (*p*s > 0.05), such as fear under fear, anger under anger, happiness under happiness, fear under neutrality, fear under sadness, sadness under disgust, fear under disgust, anger under disgust, sadness under anger, fear under anger, the second fear under fear, the second anger under anger, the second happiness under happiness, the second fear under sadness, the second sadness under disgust, the second fear under disgust, the second anger under disgust, the second sadness under anger, the second anger under surprise, the second fear under happiness. Some negative weak microexpression recognition accuracies were obstructed by strong negative backgrounds, which might be because they had a similar emotional valence. Only weak fear and anger microexpression recognition accuracies were obstructed by the strong happiness and surprise backgrounds, which might be because they had a similar face muscle status.

### Retest Reliability and Training Effect

We analyzed the correlation between weak microexpression recognition accuracies in the two measurements and found that surprise under disgust, in the two experiments, were not related (*p* > 0.05); other weak microexpression recognition accuracies in the two experiments were significantly positively related (*p* < 0.01), indicating that the WEMERT had good retest reliability (see Table 1).

We conducted a paired sample *t-*test between the two experiments and found that some weak microexpression recognition accuracies, in the second measurement, were significantly higher than those in the first measurement, such as sadness under surprise, sadness under disgust, surprise under happiness, surprise under disgust, which resulted in a training effect (see Table 1). Although the second fear under surprise measurement was significantly lower than the first, both were not higher than random measurement.

### Criterion Validity

Since there was a training effect in the second measurement, only the weak microexpression recognition accuracies in the first measurement were used as indicators to measure participants’ ability of weak microexpression recognition. The weak microexpression recognition accuracies under neutral backgrounds, which belonged to JACBART, were adopted as in the first criterion. Approximate common expression recognition accuracies were adopted as the second criterion. We conducted a Persons correlation to test criterion validity.

Weak fear microexpressions: (1) fear under happiness was significantly positively related to fear under neutrality, *r* = 0.22, *p* < 0.05; fear under anger was significantly positively related to fear under neutrality, *r* = 0.54, *p* < 0.01; (2) fear under surprise were significantly positively related to fear under fear; *r* = 0.22, *p* < 0.05; fear under happiness was significantly negatively related to fear under fear; *r* = -0.31, *p* < 0.01; (3) fear under neutrality was not related to fear under fear, *p* > 0.05.

Weak sadness microexpressions: (1) sadness under disgust, anger, surprise, and happiness were significantly positively related to sadness under neutrality, the correlation coefficient *r*s were 0.26, 0.21, 0.42 and 0.29, *p*s < 0.01; (2) sadness under fear and surprise were significantly negatively related to sadness under sadness, the *r* was -0.33 and -0.28, *p*s < 0.01; sadness under happiness was significantly negatively related to sadness under sadness, *r* = -0.20, *p* < 0.05; (3) sadness under neutrality was not related to sadness under sadness, *p* > 0.05.

Weak disgust microexpressions: (1) disgust under sadness, surprise, and happiness was significantly positively related to disgust under neutrality, and the *r*s were 0.57, 0.45 and 0.39, *p*s < 0.01; disgust under fear was significantly positively related to disgust under neutrality, *r* = 0.24, *p* < 0.05; (2) no weak disgust microexpression except disgust under neutrality was related to disgust under disgust, *p*s > 0.05; (3) disgust under neutrality was significantly positively related to disgust under disgust, *r* = 0.21, *p* < 0.05.

Weak anger microexpressions: (1) anger under sadness, fear, surprise, and happiness were significantly positively related to anger under neutrality, the *r*s were 0.30, 0.42, 0.40 and 0.47, *p*s < 0.01; (2) anger under disgust was positively related to anger under anger, *r* = 0.49, *p* < 0.05; angry under sadness and happiness were significantly negatively related to anger under anger, the *r* were -0.31 and -0.26, *p*s < 0.01; anger under surprise was significantly negatively related to anger under anger, *r* = -0.23, *p* < 0.05; (3) anger under neutrality was not related to anger under anger, *p* > 0.05.

Weak surprise microexpressions: (1) surprise under sadness, disgust, anger, and happiness were significantly positively related to surprise under neutrality, the *r*s were 0.61, 0.57, 0.60 and 0.64, *p*s < 0.01; (2) surprise under sadness, disgust, anger, and happiness were significantly negatively related to surprise under surprise, the *r*s were -0.56, -0.50, -0.48 and -0.46, *p*s < 0.01; (3) surprise under neutrality was significantly negatively related to surprise under surprise, *r* = -0.48, *p* < 0.01.

Weak happiness microexpressions: (1) happiness under sadness, disgust, fear, anger, and surprise were significantly positively related to happiness under neutrality, the *r*s were 0.70, 0.62, 0.60, 0.72 and 0.60, *p*s < 0.01; (2) happiness under sadness, disgust, fear anger and surprise were significantly negatively related to happiness under happiness, the *r*s were -0.60, -0.55, -0.64, -0.63, and -0.70, *p*s < 0.01; (3) happiness under neutrality was significantly negatively related to happiness under happiness, *r* = -0.68, *p* < 0.01.

In summary, the weak microexpression recognition accuracies under other backgrounds were universally positively related to the weak microexpression recognition accuracies under a neutral background, which proved that the weak microexpression recognition test in the current study had good calibration validity. But they were not completely related to the weak microexpression recognition accuracies under the neutral background, indicating the existence of ecological validity. A variance analysis was performed to obtain ecological validity. Some weak microexpressions under other backgrounds were positively related to the approximate common expressions, but weaker microexpressions under other backgrounds were negatively or not related to the approximate common expressions, which proved that they were more competitive and independent.

### One of the Ecological Validities: The Background Effect

An ANOVA and pairwise comparison can reveal the ecological validity. The criterion validity analysis found that other weak microexpression recognition accuracies were negatively related to approximate common expressions recognition accuracies, which already reflected ecological validity of the approximate common expressions. In order to avoid the approximate common expressions enlarging the disparity of weak microexpressions, thus exaggerating the ecological validity, we removed the approximate common expressions and then conducted a variance analysis for the first measurement, to detect the main effect of the backgrounds and to obtain the strict ecological validity.

Repeated variance analysis of fear under different backgrounds was conducted. A sphericity test showed that the variance was not homogeneous, *p* < 0.05, we then did a Greenhouse correction and found that the backgrounds main effect was significant, *F*(4.47,92) = 2.69, *p* < 0.05, $\eta_{p}^{2}$ = 0.027. The paired comparison of the Bonferroni correction showed that fear under surprise was lower than that under happiness (*p* < 0.01) and disgust (*p* < 0.05).

Repeated variance analysis of sadness under different backgrounds was conducted. A sphericity test showed that the variance was not homogeneous, *p* < 0.05, we then did a Greenhouse correction and found that the backgrounds main effect was significant, *F*(4.45,92) = 12.26, *p* < 0.01, $\eta_{p}^{2}$ = 0.112. The paired comparison of the Bonferroni correction showed that sadness under neutrality was higher than that under disgust, anger and surprise (*p*s < 0.01), but was lower than that under happiness (*p* < 0.05); sadness under happiness was higher than that under surprise (*p* < 0.01); sadness under fear was higher than that under disgust and anger (*p*s < 0.05).

Repeated variance analysis of disgust under different backgrounds was conducted. A sphericity test showed that the variance was homogeneous, *p* > 0.05. The backgrounds main effect was significant, *F*(5,92) = 15.87, *p* < 0.01, $\eta_{p}^{2}$ = 0.141. The paired comparison of the Bonferroni correction showed that disgust under neutrality was higher than that under other backgrounds (*p*s < 0.01); disgust under sadness and happiness were higher than that under anger (*p*s < 0.05); disgust under happiness and sadness were higher than that under surprise (*p*s < 0.05).

Repeated variance analysis of anger under different backgrounds was conducted. A sphericity test showed that the variance was not homogeneous, *p* < 0.05, we then did a Greenhouse correction and found that the backgrounds main effect was significant, *F*(4.16,92) = 6.07, *p* < 0.01, $\eta_{p}^{2}$ = 0.059. The paired comparison of the Bonferroni correction showed that anger under disgust was lower than those under other backgrounds (*p*s < 0.01).

Repeated variance analysis of surprise under different backgrounds was conducted. A sphericity test showed that the variance was not homogeneous, *p* < 0.05, we then did a Greenhouse correction and found that the backgrounds main effect was significant, *F*(3.91,92) = 52.11, *p* < 0.01, $\eta_{p}^{2}$ = 0.349. The paired comparison of the Bonferroni correction showed that surprise under neutrality was lower than those under other backgrounds (*p*s < 0.01); surprise under sadness was higher than those under happiness (*p* < 0.05), fear and anger (*p*s < 0.01); surprise under happiness was higher than that that under fear (*p* < 0.01) and anger (*p* < 0.05), but lower than that under disgust (*p* < 0.01); surprise under disgust and anger was higher than that under fear (*p*s < 0.01); surprise under disgust was higher than that under anger (*p* < 0.01).

Repeated variance analysis of happiness under different backgrounds was conducted. A sphericity test showed that the variance was not homogeneous, *p* < 0.05, we then did a Greenhouse correction and found that the backgrounds main effect was significant, *F*(4.16,92) = 11.15, *p* < 0.01, $\eta_{p}^{2}$ = 0.103. The paired comparison of the Bonferroni correction showed that happiness under neutrality was higher than those under other backgrounds (*p*s < 0.05); happiness under surprise was higher than that under sadness (*p* < 0.05); happiness under anger was higher than that under sadness, disgust (*ps* < 0.01) and fear (*p* < 0.05).

The paired comparison of the Bonferroni correction showed that sadness under sadness was higher than that under disgust, anger (*p*s < 0.01) and surprise (*p* < 0.05); disgust under disgust was higher than that under anger (*p* < 0.01), but was lower than that under neutrality (*p* < 0.01); fear under fear was higher than that under surprise (*p* < 0.05); anger under anger was lower than that under neutrality (*p* < 0.05) and fear (*p* < 0.01); surprise under surprise was lower than that under neutrality, sadness, disgust, and anger (*p*s < 0.01); happiness under happiness was lower than those under other backgrounds (*p*s < 0.01). With the exception of sadness, all approximate common expression recognition accuracies were not higher than other weak microexpression recognition accuracies, which showed that because there was just a small change in each approximate common expression, participants recognized them with more difficultly and incorrectly judged them as other weak microexpressions.

In summary, the backgrounds main effect of all weak microexpressions were significant, pairwise comparisons showed that there were a wide range of differences between weak microexpressions under different backgrounds. These results indicated that the weak microexpression recognition test had good ecological validity.

In the same background, there were generally significant differences among different weak microexpressions, their main effects were all significant. Generally speaking, happiness and surprise were the biggest, disgust was the second, anger was the third, and fear and sadness were the lowest. This might be because happiness and surprise had obvious characteristics- mouth open, while disgust and anger carried a high threat, and fear and sadness carried a low threat. Because the significant weak microexpressions main effect under the same background expression was common sense, it was not the focus of the current study, and no further statistical analysis was conducted. If readers are interested, refer to Table 1.

### The Second Ecological Validity: Fluctuations of Ecological Microexpression Recognition

Can the backgrounds main effect of weak microexpressions be quantified? The standard deviation of the same weak microexpression recognition accuracies under different backgrounds was defined as the fluctuation of microexpression recognition, which was the quantification index of backgrounds effect.

We did a single sample *t*-test of 0, with the standard deviation of the weak microexpression recognition accuracies in the first measurement, and found that they were all significantly greater than 0 (*p*s < 0.05), which indicated that there were significant fluctuations and that the weak microexpression recognition test had good ecological validity. Pearson’s correlation statistical analysis found that O1 (fantasy openness) was significantly positively related to the standard deviation of the weak anger microexpression recognition accuracies, O6 (value openness) was significantly negatively related to the standard deviation of the weak happiness microexpression recognition accuracies, which indicated that the two standard deviations might be personality characteristics (see Table 2).

**Table 2:** The standard deviation of the weak microexpression recognition accuracies and their relationship in the first measurement.

Weak	The first	One sample *t*-test	Cohen’s *d*	Correlation	Correlation
microexpressions	measurement	*t*(*df* = 97)		with O1	with O6
	(*M* ±*SD*)				
Sadness	0.15 ± 0.07	21.46^∗∗∗^	2.14	–	–
Disgust	0.17 ± 0.08	22.24^∗∗∗^	2.13	–	–
Fear	0.13 ± 0.08	16.87^∗∗∗^	1.63	–	–
Anger	0.15 ± 0.07	21.55^∗∗∗^	2.14	0.21^∗^	–
Surprise	0.22 ± 0.07	28.90^∗∗∗^	3.14	–	–
Happiness	0.17 ± 0.07	22.25^∗∗∗^	2.43	–	-0.20^∗^

*^∗^*p* < 0.05, ^∗∗∗^*p* < 0.001.*

### The Relationship Between Weak Microexpression Recognition With the Openness

Pearson’s correlation statistical analysis found that: (1) openness was significantly positively related to surprise under happiness and anger, the *r*s were both 0.24, *p*s < 0.05; (2) O1 was significantly negatively related to surprise under neutrality, *r* = -0.25, *p* < 0.05, but was significantly positively related to anger under surprise and surprise under happiness, the *r*s were 0.21 and 0.23, *p*s < 0.05, and was significantly positively related to anger under fear and surprise under anger, the *r*s were 0.29 and 0.28, *p*s < 0.01; (3) O2 (aesthetics openness) was significantly positively related to surprise under happiness, *r* = 0.21, *p* < 0.05; (4) O3 (feelings openness) was significantly positively related to fear under surprise and sadness under happiness and surprise under happiness, and the *r*s were 0.23, 0.22 and 0.21, *p*s < 0.05; (5) O5 (ideas openness) was significantly positively related to sadness under surprise, *r* = 0.21, *p* < 0.05.

## Discussion

### The Weak Ecological Microexpression Recognition Test (WEMERT) Had Good Reliability and Validity

The weak microexpression recognition accuracies in the two experiments were generally correlated, provided that the test had good retest reliability. Many weak microexpressions under other backgrounds were positively related to weak microexpressions under neutral backgrounds which belonged to JACBART microexpressions, provided that the test had good criterion validity. Many weak microexpressions under other backgrounds were negatively related to approximate common expressions, proving that approximate common expressions were different from other weak microexpressions and had good ecological validity. The backgrounds main effects of all weak microexpressions were significant and pairwise comparisons showed that there were a wide range of differences among weak microexpressions under different backgrounds. There were significant fluctuations in all weak microexpression recognitions, provided that the test had good ecological validity. In terms of retest reliability, criterion validity and ecological validity, it can be concluded that, the current study created a standard WEMERT which can measure weak ecological microexpression recognition stably and effectively.

The weak microexpression recognition test established by the current study includes six basic microexpressions under all seven basic expressions backgrounds, making it more ecologically valid than the JACBART by Ekman (1993) and the METT (the microexpression training tool) by Ekman (2002, 2003), whose microexpressions were only under neutral backgrounds. While Zhang et al. (2014) detected sadness and happiness microexpressions under sadness, neutral and happiness expressions, with a certain ecological validity, the backgrounds and the microexpressions did not include all the basic expressions; and because its purpose was not to establish a microexpression recognition test, no reliability or validity tests were conducted. Zhang et al. (2017) used seven high intensity basic expressions as the backgrounds, six high intensity basic expressions embedded in the backgrounds as microexpressions to establish the EMERT, but they did not detect the intensity factor of the backgrounds and the microexpressions. The current study established the weak microexpression recognition test for the first time. It was one type of the EMERT, therefore it could be called WEMERT (the weak ecological microexpression recognition test). In the future, it can be used to improve the existing METT to obtain more ecological validity of the micro expression recognition training.

### The Reliability and Validity Tests Revealed the Characteristics of Weak Ecological Microexpression Recognition

Zhang et al. (2017) found that in EMERT, all ecological microexpression recognition accuracies in two experiments were higher than random expressions. But in the current study, some of the weak microexpression recognition accuracies were not higher than random expressions, which might because of high intensity backgrounds that obstructed low intensity microexpression recognition more than high intensity microexpression recognition in Zhang et al. (2017) study. Zhang et al. (2017) found many training effects, but the current study only found training effects, which might be because weak microexpression recognition training was more difficult.

Zhang et al. (2017) found that in EMERT, ecological microexpression recognition accuracies under other backgrounds were generally positively related to both microexpressions under neutral and common expressions. However, in the current study, we found that in WEMERT, weak microexpression recognition accuracies under other backgrounds were generally positively related to weak microexpressions under neutral backgrounds but were generally negatively related to approximate common expressions. It might be because when high intensity backgrounds and low intensity microexpression were of the same expression type, the contrast was not strong enough for participants to recognize the special weak microexpression, but participants might sense a difference and ultimately judge them incorrectly as another microexpression, when they are in fact of the same expression type as the background. In EMERT, common expressions had no change, so participants could more correctly recognize them (Zhang et al., 2017).

Zhang et al. (2014) found that when the background expression was negative, all microexpression recognition accuracies were lower than that under positive or neutral backgrounds, which indicated that negative backgrounds obstructed microexpression recognition. When the target microexpressions had the same properties as the background (both positive or both negative), the microexpression recognition accuracies were significantly lower than the accuracies when the two were inconsistent. However, Zhang et al. (2017) found that the Zhang et al. (2014) study was a special case of ecological microexpression recognition. In the Zhang et al. (2017) study, some negative backgrounds also obstructed certain microexpression recognitions, but more negative backgrounds promoted certain microexpression recognitions. In some cases, when backgrounds and microexpressions had the same properties, the accuracy was lower than that when the two had different properties, but in other cases, the accuracy was higher than that when the two had different properties. For instance, fear under disgust was higher than that under surprise; sadness under fear was higher than that under surprise and happiness; sadness under disgust was higher than that under surprise and happiness; sadness under anger was higher than that under surprise; disgust under fear and under anger were higher than that under surprise.

The current study found: (1) weak microexpression recognition accuracies were between 0.12 and 0.72, widely lower than the microexpression recognition accuracies between 0.19 and 0.90 in the Zhang et al. (2017) EMERT study. This was because high intensity backgrounds damaged the low intensity microexpression recognition more in WEMERT. The backgrounds main effects of all weak microexpressions were significant. Those results were different that the Zhang et al. (2017) EMERT study, in which the backgrounds main effects of surprise and happiness were not significant. It is clear that WEMERT had higher ecological validity than EMERT, which might be because in EMERT, the high intensity surprise and happiness microexpressions were easily recognized from all the high intensity backgrounds, and the ceiling effect existed. There was too much difference between WEMERT and EMERT in paired comparison results to show all details, therefore we only discuss the main differences. Readers obtain further details of these differences in the Zhang et al. (2017) study. The current study used the standard deviation of the same microexpression recognition under different backgrounds to define fluctuations of weak microexpression recognition. It was found that the standard deviations of the weak microexpression recognition in the first and second experiments were all significantly greater than 0, which indicated that there were many fluctuations in weak microexpression recognition. Those results were the same as Zhang et al. (2017), (2) except that surprise under happiness was lower than that under sadness and disgust and all other weak microexpression recognition accuracies under positive backgrounds were not lower than those under negative backgrounds. The high intensity negative backgrounds damaged the weak microexpression recognition more than the high intensity positive backgrounds. This might be because high intensity negative backgrounds received more attention than the positive backgrounds and directed more attention away from weak microexpressions. These results were different to Zhang et al. (2017), in which many negative backgrounds promoted microexpression recognition more than the positive backgrounds (see the previous paragraph), and because the microexpression intensity was also high, it would create a strong contrast with the high intensity negative backgrounds which received more attention. (3) In many cases, when backgrounds and microexpressions had the same properties, the accuracy was lower than when the two had different properties; but just in a few cases, the accuracy was higher when the two had different properties. For instance, disgust under sadness was higher than that under surprise; surprise under happiness was higher than that that under fear and anger; happiness under surprise was higher than that under sadness. But those results were different with Zhang et al. (2017). In their study, when backgrounds and microexpressions had the same properties, in many cases, the accuracy was higher than that when the two had different properties (see the previous paragraph). (4) In summary, it is clear that by using EMERT as a criterion, WEMERT had good discrimination validity. But similar to EMERT, the relationship between weak microexpressions and backgrounds was complex in WEMERT. The relationship was either positive or negative. There was start, contrast, masking, interference, distortion, etc., so we could not use positive, negative, or consistency to sum up the relationship between them. Instead, we needed to analyze them separately to further explore their own mechanisms.

### Personality Factors of Ecological Microexpression Recognition

Mill et al. (2009) and Hurley et al. (2014) found that personality openness was positively related to some microexpression recognition under neutral in JACBART. Because the current study established the WEMERT for the first time, it found that personality openness and its sub dimensions, such as O1, O2, O3 and O5, were also positively related to some weak microexpression recognition. The exceptional thing was that in the current study, O1 was significantly negatively related to surprise under neutrality. These mechanisms should be studied further.

O1 was positively related to the standard deviation of the weak anger microexpression recognition accuracies in the first measurement, O6 was negatively related to the standard deviation of the weak happiness microexpression recognition accuracies in the first measurement, which indicated that the two standard deviations might be personality characteristics. Zhang et al. (2017) found that personality openness O, was positively correlated with the standard deviation of sadness microexpression recognition and was negatively correlated with the standard deviation of happiness microexpression recognition. Comparing the two studies, the sub dimensions of openness could more sensitively detect the fluctuations of weak microexpression recognition.

### Artificial Intelligence Recognition Based on Specific Algorithms

It is evident that as nuanced micro expressions are introduced into increasingly more sophisticated studies, detection is by humans as well as machines is more difficult. Other imaging modalities, beyond visual imaging, could offer a way to increase discriminability. Perhaps humans cannot accurately recognize such complex microexpressions and we must therefore resort to artificial intelligence. Some studies can serve as a starting point for researchers to explore, such as the comparative analysis of thermal and visual modalities for automated facial expression recognition (Wesley et al., 2012). They present a comparative analysis of performance of automated facial expression recognition from thermal facial videos, visual facial videos, and their fusion. These experimental results depict that the thermal imaging modality outperforms visual modality. The thermal imaging modality and the visual modality can also be viewed as artificial intelligence recognition. With the development of research, increasingly complex microexpressions will be found. These microexpressions can also be viewed as a form of big data. In the future, researchers should use artificial intelligence recognition based on specific algorithms.

## Conclusion

The current study created a standard WEMERT, including six weak basic microexpressions under seven high intensity basic backgrounds. The results found: (1) the test had good retest reliability, criterion validity and ecological validity; (2) the reliability and validity tests revealed many characteristics of weak microexpression recognition. There were training effects in some weak microexpression recognition. Weak microexpression recognition was generally positively related to microexpression recognitions of JACBART but were generally negatively related to approximate common expressions. The backgrounds main effects in all weak microexpressions were significant, pairwise comparisons show there were a wide range of differences between weak microexpressions under different backgrounds. The standard deviations of the accuracy of the weak microexpressions in different backgrounds were used to define the fluctuations of the weak microexpression recognition and we found that weak microexpression recognition had many fluctuations; (3) personality openness and its sub dimensions (O1, O2, O3, and O5) were generally positively related to some weak microexpression recognition, except that O1 was significantly negatively related to surprise under neutrality. O1 was positively related to the standard deviation of the weak anger microexpression recognition accuracies, and O6 was negatively related to the standard deviation of the weak happiness microexpression recognition accuracies in the first measurement.

## Author Contributions

MY was responsible for programming, data statistics and article writing, and participated in experimental design. LT assisted to revise the article and correct the English grammar. JZ provided ideas and took charge of experimental design and statistical design. WH was responsible for experimental implementation and data collection. DL was responsible for guiding the whole process, reviewing and guiding the research ethics, theories and feasibility, reviewing and revising articles, and providing financial support.

## Conflict of Interest Statement

The authors declare that the research was conducted in the absence of any commercial or financial relationships that could be construed as a potential conflict of interest.
